# The impact of Charlson Comorbidity Index on surgical complications and reoperations following simultaneous bilateral total knee arthroplasty

**DOI:** 10.1038/s41598-023-33196-x

**Published:** 2023-04-15

**Authors:** Kun-Han Lee, Wei-Lin Chang, Shang-Wen Tsai, Cheng-Fong Chen, Po-Kuei Wu, Wei-Ming Chen

**Affiliations:** 1 Department of Orthopaedics and Traumatology, Taipei Veterans General Hospital, No. 201, Sec. 2, Shi-Pai Road, Beitou District, Taipei, 112 Taiwan; 2 Department of Orthopaedics, School of Medicine, National Yang Ming Chiao Tung University, Taipei, Taiwan

**Keywords:** Medical research, Outcomes research

## Abstract

Simultaneous bilateral total knee arthroplasty (TKA) might be associated with higher postoperative morbidity and mortality rates compared with staged bilateral TKA. However, risk factors for surgical complications and reoperations following simultaneous bilateral TKA remain elusive. We conducted this retrospective, single-surgeon case series from 2010 through 2019. A total of 1561 patients who underwent simultaneous bilateral TKA procedures were included. The outcome domains included 30-day and 90-day readmission events for medical or surgical complications and 1-year reoperation events. We performed logistic regression analysis and backward stepwise selection to identify possible risk factors, including age, sex, body mass index, diabetes mellitus (DM), rheumatoid arthritis, American Society of Anesthesiologist (ASA) classification, Charlson Comorbidity Index (CCI), receiving venous thromboembolism (VTE) prophylaxis, or blood transfusion. The overall 30-day, 90-day readmission, and 1-year reoperation rates were 2.11%, 2.88%, and 1.41%, respectively. Higher CCI score (CCI = 4+) was a risk factor for 90-day readmission (aOR: 2.783; 95% CI 0.621–12.465), 90 day readmission for surgical complications (aOR: 10.779; 95% CI 1.444–80.458), and 1 year reoperation (aOR: 4.890; 95% CI 0.846–28.260). Other risk factors included older age, higher ASA level, DM, and receiving VTE prophylaxis. In conclusion, high CCI scores were associated with increased risks of surgical complications and reoperations following simultaneous bilateral TKA procedures.

## Introduction

Total knee arthroplasty (TKA) is an effective and safe procedure for clinical improvement and pain relief in patients diagnosed with osteoarthritis^1^. Considering the growth of the aging population and geriatric society, the demand of primary TKA has increased substantially^2^. Since the prevalence of bilateral knee osteoarthritis is high in the elderly population, simultaneous bilateral TKA is considered a feasible treatment option^3,4^. Simultaneous bilateral TKAs account for approximately 4% of all TKAs in the United States, or approximately 30,000 cases per year^5^.

Potential benefits of simultaneous bilateral TKAs including decreased length of hospital stay, shorter rehabilitation period, and reduced medical expenses; however, concerns remain regarding higher medical morbidity and mortality rates compared with staged bilateral TKAs, especially for those with multiple comorbidities^6–8^. Charlson Comorbidity Index (CCI), a weighted scale developed for predicting mortality risk based on categories of comorbidities in patients, has been proposed to estimate morbidity or mortality following major orthopedic surgery^9–12^. Nonetheless, although one study has proposed that CCI could be a predictor of medical complications in patients receiving simultaneous bilateral TKAs, the correlation between CCI and postoperative surgical complications and reoperations is still unclear^13^.

Asides from medical complications, surgical complications and reoperations following total joint arthroplasty procedures could have an impact on clinical outcomes and patient satisfaction^14,15^. Only a few studies have described surgical complications and reoperations following simultaneous bilateral TKA procedures; furthermore, none have validated the surgical complications with CCI or associated risk factors^16,17^.

Therefore, in this single-surgeon case series, we aim to validate the (1) rates of 30-day, 90-day readmission, and 1-year reoperation events, and (2) impact of CCI and possible risk factors for the above events following simultaneous bilateral TKA procedures.

## Materials and methods

### Data collection

**Figure 1:**
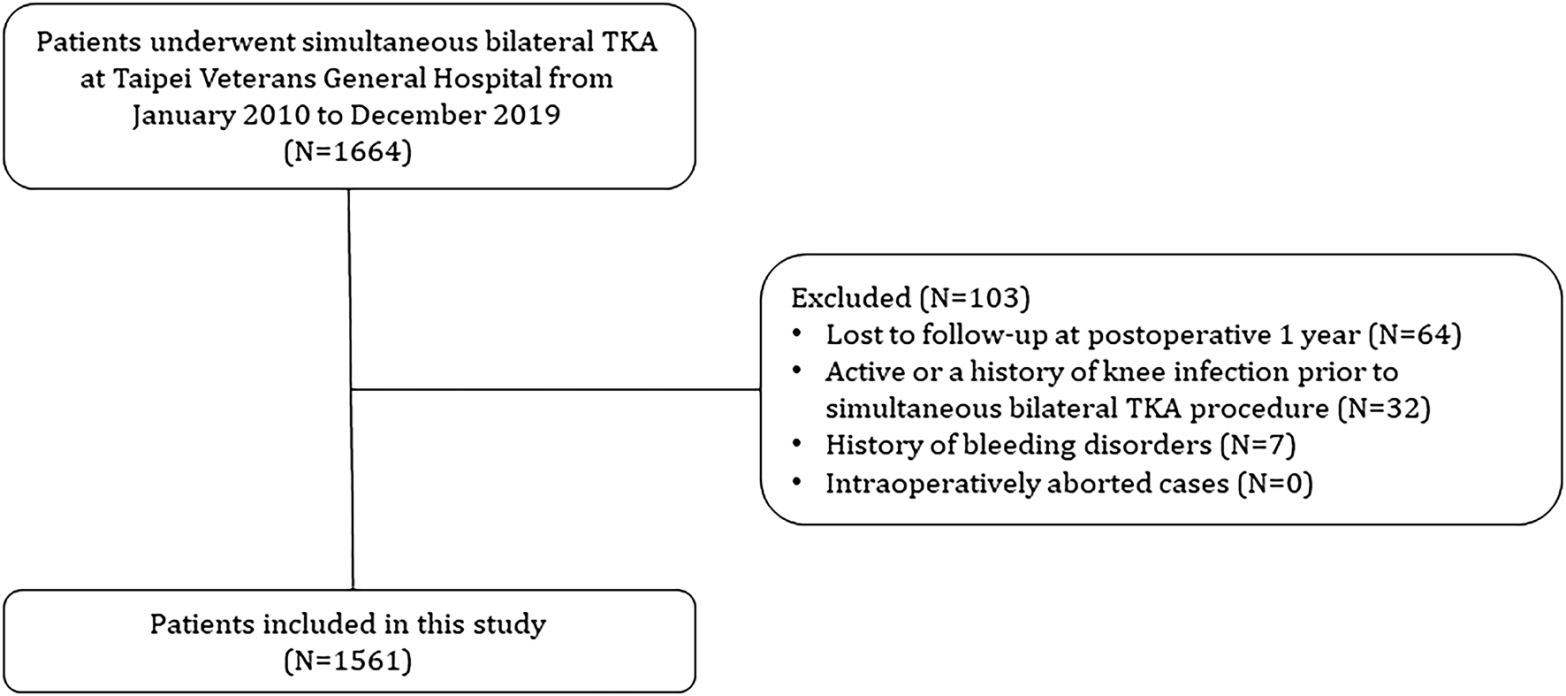
Inclusion flowchart. Abbreviation: TKA, total knee arthroplasty.

This was a retrospective, single-surgeon case series study conducted in Taipei Veterans General Hospital, a tertiary referral hospital in Taipei, Taiwan. The study was approved by the Institutional Review Board of Taipei Veterans General Hospital (2019-09-009CC). Due to the retrospective nature of the study, the Institutional Review Board of Taipei Veterans General Hospital has also approved our request to waive the inform consent documentation. All research was performed in accordance with the Declaration of Helsinki and relevant regulations. Medical records were collected between January 2010 and December 2019 from Taipei Veterans General Hospital Orthopedics database. Patients who possibly met the inclusion criteria were screened using Taiwan’s National Health Insurance procedure codes: “PCS-64169B, TKA”. Indications for primary TKA procedures included primary or secondary knee osteoarthritis (ICD-10-CM code: M17), spontaneous osteonecrosis of the knee (SONK, ICD-10-CM code: M90.55, M90.56), and rheumatoid arthritis of knee joint (RA, ICD-10-CM code: M05.76, M05.86, M06.86, M06.9). Patients who underwent simultaneous bilateral primary TKA were then selected and documented. We did not use a screening form or a predetermined patient selection protocol for simultaneous bilateral TKA procedure. The exclusion criteria were as follows: (1) lost to follow-up at postoperative 1 year; (2) active or a history of knee infection prior to simultaneous bilateral TKA procedure; and (3) history of bleeding disorders (e.g., hemophilia). The inclusion flowchart is presented in detail in Fig. 1.

Two authors (KHL and WLC) reviewed all medical records. Baseline characteristics, including age, sex, height, weight, body mass index (BMI), diabetes mellitus (DM), rheumatoid arthritis (RA), American Society of Anesthesiologist (ASA) physical status classification, CCI, receipt of venous thromboembolism (VTE) prophylaxis or blood transfusion, length of stay, and in-hospital mortality were recorded.

### Surgical technique and post-operative care protocols

All bilateral TKA procedures were conducted by a single senior orthopedic surgeon (WMC) through a minimally invasive mid-vastus approach under general anesthesia^18^. There were no intraoperatively aborted simultaneous bilateral TKA procedures because of patient or surgeon factors. The types of the TKA prostheses included Nexgen (Zimmer Biomet, Warsaw, IN, USA), NRG (Stryker, Mahwah, NJ, USA), and Triathlon (Stryker, Mahwah, NJ, USA). All TKA components were fixed with cement. A tourniquet and a close-suction drain were used in every TKA procedure. A single dose of intra-articular tranexamic acid was given after wound closure. If the patient presented with signs of hypovolemia in operating room or post-anesthesia care unit secondary to excessive intraoperative blood loss or drainage amount, packed red blood cell was transfused based on surgeon and doctor judgments.

Pharmacologic VTE prophylaxis was administered in patients with BMI ≥ 30 (kg/m^2^), presence of varicose veins, or a history of VTE events during 2010 to 2017. However, in December 2017, a patient underwent simultaneous bilateral TKA who did not meet the criteria of VTE prophylaxis mentioned above but developed pulmonary embolism during the perioperative period. Therefore, every patient who received simultaneous bilateral TKA were indicated for pharmacologic VTE prophylaxis since January 2018^19^. Intravenous prophylactic antibiotics with cefazolin were administered for 24 hours after the TKA procedure unless there was evidence of infection. All patients started ambulation and rehabilitation protocols on postoperative day 1. Hemoglobin level was also checked on the same day. If the hemoglobin level was < 9.0 g/dL or between 9.0 and 10.0 g/dL with symptoms of anemia, such as malaise, dizziness, hypotension or tachycardia, patients would receive a blood transfusion with 1–2 units of packed red blood cells.

### Outcome domains

We aimed to identify the risk factors for 30- and 90-day readmissions and 1-year reoperations following simultaneous bilateral TKA. The incidence of readmission was recorded and categorized as medical or surgical complications. Medical complications were unexpected end organ damage or systemic injury in relation to patients’ underlying status and postoperative condition, including cerebral (stroke, transient ischemic attack), cardiac (acute myocardial infarction, arrhythmia), pulmonary (respiratory failure, pneumonia), abdominal (gastric ulcer, gastrointestinal bleeding), genitourinary (acute kidney injury, urinary tract infection), coagulation (pulmonary embolism, deep vein thrombosis), or systemic (systemic inflammatory response syndrome, sepsis) problems. The surgical complications included surgical site complications (SSC), periprosthetic joint infection (PJI), patella tendon rupture, peri-prosthetic fracture, instability, quadricep tendon rupture, and any other sequelae related to surgery itself. All reoperations for surgical complications were recorded within 1 year after the index simultaneous bilateral TKA procedure.

### Statistical analysis

Statistical analyses were performed using SPSS v.25 (IBM Corp., Armonk, NY, USA) and R Statistical Software v4.2.2. (R Core Team, Vienna, Austria) Descriptive statistics were calculated for all available data. The Chi-square test or Fisher’s exact test was used for comparing discrete variables where appropriate. Univariate or multivariate logistic regression analysis was conducted to determine possible risk factors for simultaneous bilateral TKA, including age, sex, BMI, DM, RA, ASA, CCI, and receiving VTE prophylaxis or blood transfusion. Variance inflation factor (VIF) stepwise variable selection was introduced to determine possible collinearity among all variables^20^. If the highest VIF value is greater than 5, indicating the existence of highly correlated variables, the variable with highest VIF will be removed. The above step will be repeated until VIF values of all remaining variables below 5. The backward stepwise regression method was then employed to choose the optimal model. Akaike information criterion (AIC) were used to assess goodness-of-fit for each of the tested models^21^. Model fits were ranked according to their AIC values, while those with the lowest AIC values were considered the optimal models. The results were expressed as an adjusted odds ratio (aOR) with a 95% confidence interval (95% CI).

## Results

### Outcome

A total of 1,561 patients who received simultaneous bilateral TKA procedures were included in our analysis. Baseline demographic characteristics of patients are listed in Table 1. There was one (0.1%) in-hospital mortality event.

**Table 1:** Patient demographics.

	Overall (N = 1561)^a^
Age (years)	71.8 ± 6.9 (range 34–91)
Sex, N (%)	
Female	1261 (80.8%)
Male	300 (19.2%)
Height (cm)	153.9 ± 7.4 (range 134.0–182.7)
Weight (kg)	67.0 ± 11.7 (range 37.5–117.3)
Body mass index (kg/m^2^)	28.2 ± 4.2 (range 17.1–48.5)
Diabetes mellitus, N (%)	
Yes	378 (24.2%)
No	1183 (75.8%)
Rheumatoid arthritis, N (%)	
Yes	29 (1.9%)
No	1532 (98.1%)
ASA Classification, N (%)	
1	490 (31.4%)
2	932 (59.7%)
3+	139 (8.9%)
Charlson comorbidity index, N (%)	
0 to 2	318 (20.4%)
3	580 (37.2%)
4+	663 (42.5%)
Pharmacological VTE prophylaxis, N (%)	712 (45.6%)
Blood transfusion, N (%)	1246 (79.9%)
Length of stay (days)	5.8 ± 1.2 (range 3–17)
In-hospital mortality, N (%)	1 (0.1%)^b^

ASA: American Society of Anesthesiologists; VTE: venous thromboembolism.

^a^Percentages may not add up to exactly 100% due to rounding.

^b^Reason of in-hospital mortality: ischemic bowel disease.

### Incidence of 30-day readmission and associated risk factors

The overall 30-day readmission rate was 2.11% (n = 33), and incidence of 30-day readmission because of medical and surgical complications was 0.7% (n = 11) and 1.4% (n = 22), respectively. Gastrointestinal bleeding (n = 5) and urinary tract infection (n = 3) were common medical complications, while SSC (n = 14) and PJI (n = 4) were common surgical complications (Table 2). For all the variables tested, analysis of VIF values were all below 5 (age = 2.2, sex = 1, BMI = 1.7, DM = 1.4, RA = 1.1, ASA = 2.2, CCI = 3, receiving VTE prophylaxis = 1.3, and blood transfusion = 1). Backward stepwise selection showed that older age (aOR: 1.101; 95% CI 1.001–1.211) and ASA = 3+ (aOR: 18.961; 95% CI 1.161–309.593) were associated with an increased risk of 30-day readmission for medical complications (Table 3). The logistic regression analyses for all registered variables are listed in Tables S1–3. The ORs listed for removed variables in the supplementary tables were the ORs at entry of the model.

**Table 2:** Incidence of postoperative readmission and reoperation events.

30-day readmission events (n = 33, 2.11%)			
Medical complications (n = 11, 0.70%)		Surgical complications (n = 22, 1.41%)	
Gastrointestinal bleeding	5	Surgical site complication	14
Urinary tract infection	3	Prosthetic joint infection	4
Anemia	1	Patella tendon rupture	1
Coronary artery disease	1	Peri-prosthetic fracture	1
Venous thromboembolism	1	Instability	1
		Quadricep tendon avulsion	1

90-day readmission events (n = 45, 2.88%)			
Medical complications (n = 18, 1.15%)		Surgical complications (n = 27, 1.73%)	
Gastrointestinal bleeding	5	Surgical site complication	17
Urinary tract infection	3	Prosthetic joint infection	4
Venous thromboembolism	3	Patella tendon rupture	2
Ischemic stroke	2	Peri-prosthetic fracture	2
Acute kidney injury	1	Instability	1
Anemia	1	Quadricep tendon avulsion	1
Coronary artery disease	1		
Congestive heart failure	1		
Pneumonia	1		

1-year reoperation events (n = 22, 1.41%)			
Prosthetic joint infection	8		
Peri-prosthetic fracture	5		
Surgical site complication	3		
Patella tendon rupture	2		
Instability	1		
Quadricep tendon avulsion	1		
Iliotibial band syndrome	1		
Soft tissue impingement	1

**Table 3:** Risk factors for postoperative readmission and reoperation events.

Outcomes and independent variables	aOR	95% CI	*P*-value
30-day readmission (medical complications)			
Age	1.101	1.001–1.211	0.048
ASA = 3+ (reference: ASA = 1)^a^	18.961	1.161–309.593	0.039
90-day readmission (all events)			
CCI = 4+ (reference: CCI = 0–2)	2.783	0.621–12.465	0.181
90-day readmission (medical complications)			
Age	1.098	1.019–1.183	0.014
DM	3.182	1.254–8.074	0.015
90-day readmission (surgical complications)			
CCI = 4+ (reference: CCI = 0–2)	10.779	1.444–80.458	0.020
1-year reoperation			
CCI = 4+ (reference: CCI = 0–2)	4.890	0.846–28.260	0.076
VTE prophylaxis	3.230	1.257–8.298	0.015

aOR, adjusted odds ratio; ASA, American Society of Anesthesiologists classification; CCI, Charlson Comorbidity Index; CI, Confidence Interval; DM, diabetes mellitus; VTE: venous thromboembolism.

^a^ASA = 2 (reference: ASA = 1): aOR (95% CI) = 5.187 (0.499–53.868), *P*-value = 0.168.

### Incidence of 90-day readmission and associated risk factors

The overall 90-day readmission rate was 2.88% (n = 45). High CCI scores were a risk factor for 90-day readmission (CCI = 4+ : aOR: 2.783; 95% CI 0.621–12.465). The incidence of 90-day readmission because of medical and surgical complications was 1.2% (n = 18) and 1.7% (n = 27), respectively. Gastrointestinal bleeding (n = 5), urinary tract infection (n = 3) and symptomatic VTE events (n = 3) were common medical complications, while SSC (n = 17) and PJI (n = 4) were common surgical complications. Older age (aOR: 1.098; 95% CI 1.019–1.183) and DM (aOR: 3.182; 95% CI 1.254–8.074) were correlated with an increased risk of 90-day readmission for medical complications. CCI = 4+ (aOR: 10.779; 95% CI 1.444–80.458) was a risk factor for 90-day readmission due to surgical complications. The logistic regression analyses for all registered variables are listed in Tables S4–6. The ORs listed for removed variables in the supplementary tables were the ORs at entry of the model.

### Incidence of 1-year reoperation and associated risk factors

There were 22 (1.41%) 1-year reoperation events, primarily due to PJI (n = 8) and periprosthetic fracture (n = 5). High CCI scores (CCI = 4+ , aOR: 4.890; 95% CI 0.846–28.260) and receiving pharmacologic VTE prophylaxis (aOR: 3.230; 95% CI 1.257–8.298) were associated with an increased risk of 1-year reoperation. The logistic regression analyses for all registered variables are detailed in Table S7. The ORs listed for removed variables in the supplementary table were the ORs at entry of the model.

## Discussion

The most important findings of this large-scale single-surgeon case series are as follows: (1) Infection and trauma events were the main reasons for early readmission and 1-year reoperation, (2) older age, higher ASA score, and DM were risk factors for early readmission due to medical complications, (3) high CCI scores were associated with increased risk of 90-day readmission for surgical complications, and (4) high CCI scores and the administration of VTE prophylaxis were risk factors for 1-year reoperation.

Despite the potential benefits of simultaneous bilateral TKA over staged bilateral TKA, such as shorter length of stay, lower medical costs, and higher patient satisfaction, the safety of the procedure is still under debate^7,8,22^. In our study, the rates of 30-day and 90-day readmission were 2.11% and 2.88%, respectively. These rates were slightly lower than recent large-scale studies^23,24^. Hart et al. included 1771 patients who underwent simultaneous bilateral TKA procedure and the 30-day readmission rate was 3.6%^23^. Wang et al. reviewed 4613 patients who received simultaneous bilateral TKA procedures as reported in the National Surgical Improvement Program database. The postoperative 30-day major complication rate was 2.59%^24^. The slightly lower readmission rate observed in our cohort (Taiwanese, Asian cohort) could be attributed to the lower incidence of symptomatic VTE. Symptomatic VTE events accounted for only 3% of the complications in our cohort, but for 38% and 60% of the complications recorded by Hart et al. and Wang et al., respectively^23,24^. The 1-year reoperation rate (1.41%) in our study was low and consistent with the rates (1.2–1.48%) in two large-scale studies reported by Bolognesi et al. and Bini et al.^17,25^ Notably, infection (including SSC and PJI) and trauma events (including fracture and tendon rupture) accounted for 86.4% of the reasons for early reoperation in our study.

Risk factors for major medical complications following simultaneous bilateral TKA procedures have been explored^26–28^. Several comorbidities, including congestive heart failure, pulmonary hypertension, chronic obstructive pulmonary disease, and renal diseases have been identified to be associated with major medical complications^26–28^. In the current study, elderly patients, higher ASA classification, and DM were associated with a higher risk of 30-day and 90-day readmission for medical complications. These findings were consistent with the results from database studies that reported older age as a risk factor for major medical complications and in-hospital mortality^26,29^. Age should be recognized as an important, non-modifiable factor when selecting patients for simultaneous bilateral TKA procedures. The finding that higher ASA levels correlated with higher risk of 30-day readmission due to medical complications is sensible since ASA is incorporated in multiple risk calculators and tools for estimating probabilities of 30-day postoperative complications and mortality^30,31^. Schaeffer et al. also reported that an ASA score ≥ 3 is associated with a 2.9 times (*P* = 0.0082) greater risk of 30-day readmission among patients receiving total joint arthroplasty^32^. On the other hand, the hyperglycemic status of DM patients could have led to immune system alterations, reduced and damaged end organ reservoirs, and increased risk of infection^33^. Patients with DM have been reported to have higher risks of congestive heart failure and renal failure after receiving surgery for hip fractures^34^. Moreover, Lovecchio et al. reported that patients with insulin-dependent DM are more likely to have medical complications or be readmitted within 30 days following total joint replacement. Interestingly, the surgical complication rate was not different between patients who had DM and those who did not in the same cohort^35^. The risk of medical complications was even higher when there was a stress hyperglycemic status before orthopedic surgeries^36^. Therefore, as a modifiable risk factor, the glucose level in patients with DM should be strictly monitored before receiving simultaneous bilateral TKA to avoid medical complications and readmissions.

Due to low rate of medical complications noted in our cohort, we then focused on addressing the issue of possible risk factors in relation to surgical complications. An important finding of this study was that high CCI scores (CCI = 4+) were associated with an increased risk of 90-day readmission for surgical complications and 1-year reoperation. Infection (including SSC and PJI) and trauma events (including fracture and extensor mechanism failure) were the main reasons for 90-day readmission due to surgical complications (96.3%, n = 26 of 27) and for 1-year reoperation (86.4%, n = 19 of 22). To the best of our knowledge, there is not a study that validates the risk factors for surgical complications following simultaneous bilateral TKA procedures. Several studies have mentioned the association between higher CCI scores and morbidity or mortality following major orthopedic surgery^10–12,37^. Moreover, higher CCI scores were correlated with low skeletal muscle mass and slower gait speed, indicating that these patients might be more fragile and sarcopenic with poorer physical performance^38^. The diseases that were included in the CCI, such as age, DM, cirrhosis, renal failure, cancer, and stroke were associated with sarcopenia, increased bone absorption, osteoporosis, increased risk of fall, and might have a negative impact on the implant survival of total joint arthroplasty^38,39^. The association between high CCI scores and infection events (including SSC and PJI) following major orthopedic procedures have been identified in several large-scale studies^40–42^. Kurtz et al. analyzed 15,674 patients who received instrumented lumbar fusion from a Medicare database. High CCI scores (CCI = 5 points) were associated with an increased risk of infection within 10 years post-surgical procedure (hazard ratio: 2.48; 95% CI 1.93–3.19)^40^. Soohoo et al. included 138,399 patients that received a total hip arthroplasty procedure from California’s statewide database. Higher CCI was associated with increased risk of several complications, including thromboembolism (OR: 1.11, 95% CI 1.04–1.19), dislocation (OR: 1.10, 95% CI 1.05–1.15), and infection (OR: 1.22, 95% CI 1.15–1.28)^42^. Similar findings were found in another statewide database study (N = 222,684 for patients receiving TKA) conducted by Soohoo et al. that higher CCI scores were associated with an increased risk of infection within 90 days after discharge^41^. As for simultaneous bilateral TKA, Marya et al. first demonstrated that both CCI (OR: 3.353, 95% CI 1.081–10.403) and age-adjusted CCI (OR: 4.165, 95% CI 1.874–9.256) are associated with major complications in patients undergoing simultaneous bilateral TKA^13^. Amit et al. also found that patients who with age-adjusted CCI scores ≥ 5 suffer from significantly higher rates of major (OR: 3.96, 95% CI 1.13–13.82) and minor (OR: 3.31, 95% CI 1.11–9.88) cumulative complications when receiving simultaneous bilateral TKA than those receiving unilateral TKA procedure^43^. The author thereby proposed that age-adjusted CCI ≥ 5 could be a feasible cut-off for selecting appropriate candidates receiving simultaneous bilateral TKA procedure. In summary, our findings are in line with previous literature reporting that CCI can be a predictive determinant for risk stratification of postoperative complications and reoperations in patients undergoing simultaneous bilateral TKA, suggesting that the burden of comorbid diseases in the practice of simultaneous bilateral TKA should be taken into consideration by both surgeons and patients for shared decision-making. However, the findings warrant further exploration and need to be validated by database registries or large-scale studies.

Pharmacological VTE prophylaxis might lead to postoperative hematoma and prolonged wound drainage, which increases wound tension, risk of dehiscence and provides a retrograde pathway for pathogens^44,45^. In our study, for the 11 patients who received reoperation because of SSC or PJI within 1 year, 10 (90.9%) received pharmacological VTE prophylaxis. This finding was consistent with our recent work that there was an association between administration of pharmacological VTE prophylaxis and risk of PJI in the early postoperative period in patients who received a total joint arthroplasty procedure (90-day PJI events, aOR: 3.2, 95% CI 1.2–8.7)^19^. As a result, the increased risk of infection correlated with pharmacological VTE prophylaxis, albeit the potential benefit of reducing VTE events should be thoroughly considered in patients receiving simultaneous bilateral TKA, especially when determining the VTE prophylaxis strategy in an Asian population who tended to have lower incidence of VTE^46^.

There were several limitations of this study. First, modifications of surgical technique and perioperative protocol over the 10-year study period could have affected the outcome domains of this study, including readmission and 1-year reoperation events. Second, the single-surgeon study design might influence the generalizability of the results. In addition, the overall readmission and reoperation rates were low in this cohort. As a result, the small number of target events might cause a wide range of confidence interval in the multivariate regression analysis. Third, the inherent limitations of the retrospective study design with only 1-year follow-up period should be recognized. Fourth, blood transfusion criteria for patients receiving simultaneous bilateral TKAs were more liberal than the American Association of Blood Banks clinical practice guidelines on blood transfusion, leading to a higher transfusion rate in our study population^47^.

## Conclusion

Simultaneous bilateral TKA appears to be a safe procedure with regard to the low rates of 30-day and 90-day readmission, as well as 1-year reoperation. High CCI scores were associated with an increased risk of surgical complications and reoperations following simultaneous bilateral TKA procedures.

## Supplementary Information

Supplementary Information 1.

Supplementary Information 2.

Supplementary Information 3.

Supplementary Information 4.

Supplementary Information 5.

Supplementary Information 6.

Supplementary Information 7.

## Data Availability

All data generated during and/or analyzed during the current study are available from the corresponding author on reasonable request.

## Abbreviations

95% CI: 95% Confidence interval
aOR: Adjusted odds ratio
ASA: American Society of Anesthesiologist
BMI: Body mass index
CCI: Charlson Comorbidity Index
DM: Diabetes mellitus
OR: Odds ratio
PJI: Periprosthetic joint infection
RA: Rheumatoid arthritis
SSC: Surgical site complications
TKA: Total knee arthroplasty
VTE: Venous thromboembolism
