# Supplementary material for: The impact of Charlson Comorbidity Index on surgical complications and reoperations following simultaneous bilateral total knee arthroplasty
Source: Sci Rep. 2023 Apr 15;13:6155. doi: 10.1038/s41598-023-33196-x (PMC10105729; doi:10.1038/s41598-023-33196-x)
Supplement: Supplementary file 1 — Supplementary Information 1. [file 41598_2023_33196_MOESM1_ESM.docx]

**Table S1.** Logistic regression analysis with backward stepwise selection of risk factors for 30-day readmission

| Variables | 30-day readmission (n=33) | No 30-day readmission  (n=1528) | Logistic regression | | Model Fitting Criteria | |
| --- | --- | --- | --- | --- | --- | --- |
|  |  |  | *P*-value | Odds ratio^a^  (95%CI) | Step of removal | AIC |
| All variables | - | - | - | - | Entered | 335.439 |
| BMI  ASA  ASA=1  ASA=2  ASA=3+ | 28.5±4.7  1.9±0.6  7 (21.2%)  22 (66.7%)  4 (12.1%) | 28.2±4.2  1.8±0.6  483 (31.6%)  910 (59.6%)  135 (8.8%) | 0.744  0.191  -  0.522  0.573 | 1.013 (0.935-1.098)  1.470 (0.825-2.618)  Reference  1.425 (0.483-4.206)  1.607 (0.309-8.367) | 1  2  -  -  - | 333.444  329.905  -  -  - |
| DM, n (%)  Blood transfusion, n (%)  Sex, n (Male %)  Age (years)  VTE prophylaxis, n (%) | 9 (27.3%)  28 (84.8%)  73.8±6.6  8 (24.2%)  17 (51.5%) | 369 (24.1%)  1218 (79.7%)  71.8±6.9  292 (19.1%)  695 (45.5%) | 0.679  0.469  0.086  0.461  0.492 | 1.178 (0.543-2.557)  1.425 (0.546-3.721)  1.047 (0.994-1.104)  1.355 (0.605-3.034)  1.273 (0.639-2.539) | 3  4  5  6  7 | 328.003  326.441  324.915  323.576  322.140 |
| RA, n (%) | 0 (0.0%) | 29 (1.9%) | 0.998 | - | 8 | 321.831 |
| CCI  CCI=0-2  CCI=3  CCI=4+ | 3.8±1.3  4 (12.1%)  9 (27.3%)  20 (60.6%) | 3.4±1.2  314 (20.5%)  571 (37.4%)  643 (42.1%) | 0.050  -  0.725  0.106 | 1.274 (1.000-1.623)  Reference  1.237 (0.378-4.050)  2.442 (0.828-7.204) | 9  -  -  - | 321.232  -  -  - |

AIC: Akaike information criterion; ASA: American Society of Anesthesiologists classification; BMI: body mass index; CCI: Charlson comorbidity index; CI**:** Confidence Interval; DM: diabetes mellitus; RA: rheumatoid arthritis; VTE: venous thromboembolism

^a^ The odds ratios listed for removed variables are those at entry of the model
