# Supplementary material for: The impact of Charlson Comorbidity Index on surgical complications and reoperations following simultaneous bilateral total knee arthroplasty
Source: Sci Rep. 2023 Apr 15;13:6155. doi: 10.1038/s41598-023-33196-x (PMC10105729; doi:10.1038/s41598-023-33196-x)
Supplement: Supplementary file 2 — Supplementary Information 2. [file 41598_2023_33196_MOESM2_ESM.docx]

**Table S2.** Logistic regression analysis with backward stepwise selection of risk factors for 30-day readmission for medical complications

| Variables | 30-day readmission with medical complications  (n=11) | No 30-day readmission with medical complications  (n=1550) | Logistic regression | | Model Fitting Criteria | |
| --- | --- | --- | --- | --- | --- | --- |
|  |  |  | *P*-value | Odds ratio^a^  (95%CI) | Step of removal | AIC |
| All variables | - | - | - | - | Entered | 133.827 |
| DM, n (%) | 4 (36.4%) | 374 (24.1%) | 0.352 | 1.797 (0.523-6.172) | 1 | 131.956 |
| RA, n (%)  Blood transfusion, n (%)  BMI | 0 (0.0%)  11 (100.0%)  28.5±.5.6 | 29 (1.9%)  1235 (79.7%)  28.2±4.2 | 0.998  0.994  0.818 | -  -  1.016 (0.886-1.166) | 2  3  4 | 130.481  129.082  128.854 |
| VTE prophylaxis, n (%)  Sex, n (Male %) | 6 (54.5%)  4 (36.4%) | 706 (45.5%)  296 (19.1%) | 0.553  0.161 | 1.435 (0.436-4.720)  2.421 (0.704-8.324) | 5  6 | 127.381  126.814 |
| CCI  CCI=0-2  CCI=3  CCI=4+  ASA  ASA=1  ASA=2  ASA=3+  Age (years) | 4.2±1.5  2 (18.2%)  1 (9.1%)  8 (72.7%)  2.2±0.6  1 (9.1%)  7 (63.6%)  3 (27.3%)  75.9±8.1 | 3.4±1.2  316 (20.4%)  579 (37.4%)  655 (42.3%)  1.8±0.6  489 (31.5%)  925 (59.7%)  136 (8.8%)  71.8±6.9 | 0.042  -  0.290  0.407  0.024  -  0.168  0.039  0.048 | 1.466 (1.013-2.121)  Reference  0.273 (0.025-3.021)  1.930 (0.407-9.141)  3.159 (1.162-8.588)  Reference  5.187 (0.499-53.868)  18.961 (1.161-309.593)  1.101 (1.001-1.211) | 7  -  -  -  -  -  -  -  - | 126.646  -  -  -  128.225  -  -  -  128.965 |

AIC: Akaike information criterion; ASA: American Society of Anesthesiologists classification; BMI: body mass index; CCI: Charlson comorbidity index; CI**:** Confidence Interval; DM: diabetes mellitus; RA: rheumatoid arthritis; VTE: venous thromboembolism

^a^ The odds ratios listed for removed variables are those at entry of the model
