# Supplementary material for: The impact of Charlson Comorbidity Index on surgical complications and reoperations following simultaneous bilateral total knee arthroplasty
Source: Sci Rep. 2023 Apr 15;13:6155. doi: 10.1038/s41598-023-33196-x (PMC10105729; doi:10.1038/s41598-023-33196-x)
Supplement: Supplementary file 3 — Supplementary Information 3. [file 41598_2023_33196_MOESM3_ESM.docx]

**Table S3.** Logistic regression analysis with backward stepwise selection of risk factors for 30-day readmission for surgical complications

| Variables | 30-day readmission with surgical complications  (n=22) | No 30-day readmission with surgical complications  (n=1539) | Logistic regression | | Model Fitting Criteria | |
| --- | --- | --- | --- | --- | --- | --- |
|  |  |  | *P*-value | Odds ratio^a^  (95%CI) | Step of removal | AIC |
| All variables | - | - | - | - | Entered | 249.364 |
| Sex, n (Male %)  VTE prophylaxis, n (%)  Blood transfusion, n (%) | 4 (18.2%)  11 (50.0%)  17 (77.3%) | 296 (19.2%)  701 (45.5%)  1229 (79.9%) | 0.901  0.678  0.764 | 0.933 (0.314-2.778)  1.195 (0.515-2.774)  0.858 (0.314-2.343) | 1  2  3 | 247.373  245.457  243.552 |
| DM, n (%)  Age (years)  BMI | 5 (22.7%)  72.8±5.7  28.4±4.4 | 373 (24.2%)  71.8±7.0  28.2±4.2 | 0.870  0.487  0.813 | 0.919 (0.337-2.509)  1.022 (0.960-1.088)  1.012 (0.917-1.116) | 4  5  6 | 241.958  240.368  238.828 |
| ASA  ASA=1  ASA=2  ASA=3+  RA, n (%) | 1.8±0.5  6 (27.3%)  15 (68.2%)  1 (4.5%)  0 (0.0%) | 1.8±0.6  484 (31.4%)  917 (59.6%)  138 (9.0%)  29 (1.9%) | 0.985  -  0.570  0.621  0.998 | 0.993 (0.489-2.018)  Reference  1.320 (0.509-3.423)  0.585 (0.070-4.896)  - | 7  -  -  -  8 | 236.040  -  -  -  234.756 |
| CCI  CCI=0-2  CCI=3  CCI=4+ | 3.6±1.1  2 (9.1%)  8 (36.4%)  12 (54.5%) | 3.4±1.2  316 (20.5%)  572 (37.2%)  651 (42.3%) | 0.339  -  0.318  0.163 | 1.164 (0.852-1.590)  Reference  2.210 (0.467-10.467)  2.912 (0.648-13.088) | 9  -  -  - | 233.218  -  -  - |

AIC: Akaike information criterion; ASA: American Society of Anesthesiologists classification; BMI: body mass index; CCI: Charlson comorbidity index; CI**:** Confidence Interval; DM: diabetes mellitus; RA: rheumatoid arthritis; VTE: venous thromboembolism

^a^ The odds ratios listed for removed variables are those at entry of the model
