# Supplementary material for: The impact of Charlson Comorbidity Index on surgical complications and reoperations following simultaneous bilateral total knee arthroplasty
Source: Sci Rep. 2023 Apr 15;13:6155. doi: 10.1038/s41598-023-33196-x (PMC10105729; doi:10.1038/s41598-023-33196-x)
Supplement: Supplementary file 4 — Supplementary Information 4. [file 41598_2023_33196_MOESM4_ESM.docx]

**Table S4.** Logistic regression analysis with backward stepwise selection of risk factors for 90-day readmission

| Variables | 90-day readmission (n=45) | No 90-day readmission  (n=1516) | Logistic regression | | Model Fitting Criteria | |
| --- | --- | --- | --- | --- | --- | --- |
|  |  |  | *P*-value | Odds ratio^a^  (95%CI) | Step of removal | AIC |
| All variables | - | - | - | - | Entered | 418.192 |
| Blood transfusion, n (%) | 36 (80.0%) | 1210 (79.8%) | 0.976 | 1.012 (0.482-2.123) | 1 | 416.202 |
| BMI  ASA  ASA=1  ASA=2  ASA=3+ | 28.4±.4.4  1.9±0.5  10 (22.2%)  31 (68.9%)  4 (8.9%) | 28.2±4.2  1.8±0.6  480 (31.7%)  901 (59.4%)  135 (8.9%) | 0.720  0.294  -  0.173  0.557 | 1.013 (0.945-1.085)  1.304 (0.794-2.144)  Reference  1.651 (0.803-3.397)  1.422 (0.439-4.606) | 2  3  -  -  - | 414.313  410.954  -  -  - |
| DM, n (%)  Sex, n (Male %)  Age (years)  VTE prophylaxis, n (%) | 15 (33.3%)  10 (22.2%)  74.1±6.2  23 (51.1%) | 363 (23.9%)  290 (19.1%)  71.7±6.9  689 (45.4%) | 0.151  0.604  0.023  0.453 | 1.588 (0.845-2.985)  1.208 (0.591-2.468)  1.054 (1.007-1.103)  1.255 (0.693-2.271) | 4  5  6  7 | 409.058  407.266  405.856  404.578 |
| RA, n (%) | 0 (0.0%) | 29 (1.9%) | 0.998 | - | 8 | 404.003 |
| CCI  CCI=0-2  CCI=3  CCI=4+ | 3.9±1.1  4 (8.9%)  12 (26.7%)  29 (64.4%) | 3.4±1.2  316 (20.7%)  568 (37.5%)  634 (41.8%) | 0.017  -  0.628  0.181 | 1.289 (1.047-1.587)  Reference  1.368 (0.385-4.865)  2.783 (0.621-12.465) | -  -  -  - | 409.868  -  -  - |

AIC: Akaike information criterion; ASA: American Society of Anesthesiologists classification; BMI: body mass index; CCI: Charlson comorbidity index; CI**:** Confidence Interval; DM: diabetes mellitus; RA: rheumatoid arthritis; VTE: venous thromboembolism

^a^ The odds ratios listed for removed variables are those at entry of the model
