# Supplementary material for: The impact of Charlson Comorbidity Index on surgical complications and reoperations following simultaneous bilateral total knee arthroplasty
Source: Sci Rep. 2023 Apr 15;13:6155. doi: 10.1038/s41598-023-33196-x (PMC10105729; doi:10.1038/s41598-023-33196-x)
Supplement: Supplementary file 5 — Supplementary Information 5. [file 41598_2023_33196_MOESM5_ESM.docx]

**Table S5** Logistic regression analysis with backward stepwise selection of risk factors for 90-day readmission for medical complications

| Variables | 90-day readmission with medical complications  (n=18) | No 90-day readmission with medical complications  (n=1543) | Logistic regression | | Model Fitting Criteria | |
| --- | --- | --- | --- | --- | --- | --- |
|  |  |  | *P*-value | Odds ratio^a^  (95%CI) | Step of removal | AIC |
| All variables | - | - | - | - | Entered | 204.923 |
| BMI | 28.5±4.8 | 28.2±4.2 | 0753 | 1.017 (0.914-1.133) | 1 | 202.923 |
| ASA  ASA=1  ASA=2  ASA=3+  Blood transfusion, n (%)  RA, n (%)  VTE prophylaxis, n (%) | 2.0±0.6  3 (16.7%)  12 (66.7%)  3 (16.7%)  14 (77.8%)  0 (0.0%)  9 (50.0%) | 1.8±0.6  487 (31.6%)  920 (59.6%)  136 (8.8%)  1232 (79.8%)  29 (1.9%)  703 (45.6%) | 0.108  -  0.618  0.555  0.828  0.998  0.707 | 1.893 (0.870-4.120)  Reference  1.498 (0.307-7.316)  1.906 (0.224-16.234)  0.884 (0.289-2.703)  -  1.195 (0.472-3.026) | 2  -  -  -  3  4  5 | 199.331  -  -  -  197.441  195.829  194.258 |
| CCI  CCI=0-2  CCI=3  CCI=4+ | 4.1±1.3  2 (11.1%)  3 (16.7%)  13 (72.2%) | 3.4±1.2  316 (20.5%)  577 (37.4%)  650 (42.1%) | 0.031  -  0.830  0.131 | 1.397 (1.032-1.891)  Reference  0.821 (0.137-4.942)  3.160 (0.709-14.088) | 6  -  -  - | 191.693  -  -  - |
| Sex, n (Male %) | 5 (27.8%) | 295 (19.1%) | 0.359 | 1.627 (0.576-4.600) | 7 | 190.416 |
| DM, n (%)  Age (years) | 9 (50.0%)  75.8±7.0 | 369 (23.9%)  71.8±6.9 | 0.015  0.014 | 3.182 (1.254-8.074)  1.098 (1.019-1.183) | -  - | 194.006  194.808 |

AIC: Akaike information criterion; ASA: American Society of Anesthesiologists classification; BMI: body mass index; CCI: Charlson comorbidity index; CI**:** Confidence Interval; DM: diabetes mellitus; RA: rheumatoid arthritis; VTE: venous thromboembolism

^a^ The odds ratios listed for removed variables are those at entry of the model
