# Supplementary material for: The impact of Charlson Comorbidity Index on surgical complications and reoperations following simultaneous bilateral total knee arthroplasty
Source: Sci Rep. 2023 Apr 15;13:6155. doi: 10.1038/s41598-023-33196-x (PMC10105729; doi:10.1038/s41598-023-33196-x)
Supplement: Supplementary file 6 — Supplementary Information 6. [file 41598_2023_33196_MOESM6_ESM.docx]

**Table S6** Logistic regression analysis with backward stepwise selection of risk factors for 90-day readmission for surgical complications

| Variables | 90-day readmission with surgical complications  (n=27) | No 90-day readmission with surgical complications  (n=1534) | Logistic regression | | Model Fitting Criteria | |
| --- | --- | --- | --- | --- | --- | --- |
|  |  |  | *P*-value | Odds ratio^a^  (95%CI) | Step of removal | AIC |
| All variables | - | - | - | - | Entered | 285.911 |
| Sex, n (Male %)  Blood transfusion, n (%) | 5 (18.5%)  22 (81.5%) | 295 (19.2%)  1224 (79.8%) | 0.926  0.828 | 0.955 (0.359-2.542)  1.114 (0.419-2.966) | 1  2 | 283.914  281.966 |
| BMI  VTE prophylaxis, n (%) | 28.4±4.2  14 (51.9%) | 28.2±4.2  698 (45.5%) | 0.840  0.512 | 1.009 (0.923-1.103)  1.290 (0.602-2.762) | 3  4 | 280.184  278.957 |
| DM, n (%) | 6 (22.2%) | 372 (24.3%) | 0.807 | 0.892 (0.358-2.228) | 5 | 277.818 |
| RA, n (%)  Age (years)  ASA  ASA=1  ASA=2  ASA=3+ | 0 (0.0%)  73.0±5.5  1.8±0.5  7 (25.9%)  19 (70.4%)  1 (3.7%) | 29 (1.9%)  71.8±7.0  1.8±0.6  483 (31.5%)  913 (59.5%)  138 (9.0%) | 0.998  0.364  0.981  -  0.417  0.518 | -  1.027 (0.970-1.087)  1.008 (0.531-1.913)  Reference  1.436 (0.599-3.440)  0.500 (0.061-4.098) | 6  7  8  -  -  - | 276.726  275.608  273.892  -  -  - |
| CCI  CCI=0-2  CCI=3  CCI=4+ | 3.7±1.2  2 (7.4%)  9 (33.3%)  16 (59.3%) | 3.4±1.0  316 (20.6%)  571 (37.2%)  646 (42.2%) | 0.121  -  0.120  0.020 | 1.461 (0.905-2.357)  Reference  3.925 (0.701-21.967)  10.779 (1.444-80.458) | -  -  -  - | 274.622  -  -  - |

AIC: Akaike information criterion; ASA: American Society of Anesthesiologists classification; BMI: body mass index; CCI: Charlson comorbidity index; CI: Confidence Interval; DM: diabetes mellitus; RA: rheumatoid arthritis; VTE: venous thromboembolism

^a^ The odds ratios listed for removed variables are those at entry of the model
