# Supplementary material for: The impact of Charlson Comorbidity Index on surgical complications and reoperations following simultaneous bilateral total knee arthroplasty
Source: Sci Rep. 2023 Apr 15;13:6155. doi: 10.1038/s41598-023-33196-x (PMC10105729; doi:10.1038/s41598-023-33196-x)
Supplement: Supplementary file 7 — Supplementary Information 7. [file 41598_2023_33196_MOESM7_ESM.docx]

**Table S7** Logistic regression analysis with backward stepwise selection of risk factors for 1-year reoperation

| Variables | 1-year reoperation  (n=22) | No 1-year reoperation  (n=1539) | Logistic regression | | Model Fitting Criteria | |
| --- | --- | --- | --- | --- | --- | --- |
|  |  |  | *P*-value | Odds ratio^a^  (95%CI) | Step of removal | AIC |
| All variables | - | - | - | - | Entered | 234.545 |
| Blood transfusion, n (%)  ASA  ASA=1  ASA=2  ASA=3+  RA, n (%)  Sex, n (Male %)  Age (years)  DM, n (%)  BMI | 17 (77.3%)  2.1±0.5  2 (9.1%)  17 (77.3%)  3 (13.6%)  0 (0.0%)  6 (23.1%)  72.0±6.9  7 (31.8%)  30.4±5.3 | 1229 (79.9%)  1.8±0.6  488 (31.7%)  915 (59.5%)  136 (8.8%)  29 (1.9%)  294 (19.2%)  71.8±6.9  371 (24.1%)  28.2±4.2 | 0.764  0.033  -  0.702  0.829  0.998  0.675  0.916  0.405  0.018 | 0.858 (0.314-2.343)  2.159 (1.066-4.373)  Reference  1.394 (0.253-7.666)  0.829 (0.077-8.891)  -  1.240 (0.454-3.389)  1.003 (0.944-1.066)  1.469 (0.595-3.631)  1.108 (1.018-1.207) | 1  2  -  -  -  3  4  5  6  7 | 232.634  229.414  -  -  -  227.805  226.302  224.919  223.386  222.590 |
| CCI  CCI=0-2  CCI=3  CCI=4+ | 4.1±1.4  3 (13.6%)  3 (13.6%)  16 (72.7%) | 3.4±1.2  315 (20.5%)  577 (37.5%)  647 (42.0%) | 0.012  -  0.777  0.076 | 1.420 (1.081-1.865)  Reference  0.776 (0.134-4.493)  4.890 (0.846-28.260) | -  -  -  - | 228.478  -  -  - |
| VTE prophylaxis, n (%) | 16 (72.7%) | 696 (45.2%) | 0.015 | 3.230 (1.257-8.298) | - | 228.308 |

AIC: Akaike information criterion; ASA: American Society of Anesthesiologists classification; BMI: body mass index; CCI: Charlson comorbidity index; CI: Confidence Interval; DM: diabetes mellitus; RA: rheumatoid arthritis; VTE: venous thromboembolism

^a^ The odds ratios listed for removed variables are those at entry of the model
